# Asylums in India

**Published:** 1893-10-21

**Authors:** 


### Asylums in India.

### IV.—THE CENTRAL PROVINCES.

**Calculated at 54 superficial feet per patient, the capacity of the Nagpur and Jubbulpore Asylums is for 162 and 163, and the maximum number of patients in confinement on any one night during 1891 was 135 and 139 respectively, showing that there was no overcrowding at either institution. The movement of population was as follows:—**

| | Nagpur. | Jubbulpore. |
|---|---|---|
| Remained on 1st January | 125 | 134 |
| Admitted and re-admitted | 29 | 29 |
| Total population | 154 | 163 |
| Discharged, cured, or improved | 11 | 14 |
| Discharged otherwise | 4 | 4 |
| Died | 8 | 15 |
| Remained on December 31st | 131 | 130 |

**In both asylums the daily average strength was higher than in the previous year, being 127·21 against 118·27 at Nagpur, and 133·77 against 130·79 at Jubbulpore. The percentage of cured to daily average strength fell at Nagpur from 6 76 to 3·93, and rose at Jubbulpore from 5·35 to 8·97. Taking the two asylums together this gives a percentage of 6 5 cured to the daily average strength of insane in the Central Provinces. The death-rate at Nagpur, calculated on the daily average strength, fell from 6·76 to 6·29, and at Jubbulpore rose from 9·94 to 11·21. The total number of criminal insane at Nagpur was 55, with a daily average strength of 46·15, and at Jubbulpore 38, with a daily average of 31·56. The financial statements show the total expenditure of the Nagpur Asylum to have been Rs.12,003 13a. 10p., and upon the Jubbulpore Asylum Rs.10,866 9a. 10p., showing the average actual cost per patient as Rs.71 14a. 2p. at Nagpur, and Rs.71 0a. 6p. at Jubbulpore. The statistics in general present no specially interesting features.**

### INSECTS AND GROWING FRUIT.

Insects play a more important part in fruit growing than cultivators generally are aware of. A Washington authority on this subject calls our attention to the necessity of cross fertilization by means of insects before the fruit will set. Pears, for instance, are amongst those fruits which for any prolific bearing require primarily to receive pollen from other varieties through the medium of the passing insect, which, in its search for honey, leaves behind it the necessary pollen, in this manner acting the part of an unconscious, but useful factor in the production of fruit.

